# A Neonicotinoid Insecticide at a Rate Found in Nectar Reduces Longevity but Not Oogenesis in Monarch Butterflies, *Danaus plexippus* (L.). (Lepidoptera: Nymphalidae)

**DOI:** 10.3390/insects10090276

**Published:** 2019-09-01

**Authors:** David G. James

**Affiliations:** Department of Entomology, Irrigated Agriculture Research and Extension Center, Washington State University, 24106 North Bunn Road, Prosser, WA 99350, USA; david_james@wsu.edu

**Keywords:** imidacloprid, neonicotinoids, adult monarch butterfly, survival, monarch butterfly decline

## Abstract

The monarch butterfly in North America has suffered a serious population decline since the mid-1990s. The introduction and widespread use of neonicotinoid insecticides during the same time period has been suggested as a potential driver of this decline but no studies have looked at the impact of these insecticides on adult monarchs. A brief laboratory study assessed the impact of Imidacloprid, the most commonly used neonicotinoid, on western monarch butterfly longevity and oogenesis. Imidacloprid at 23.5 ppb, a field-realistic rate reported from wild nectar and pollen, was fed ad libitum to newly-eclosed monarchs in a sugar-based diet for 22 days. Treated monarchs showed reduced longevity, suffering 78.8% mortality by day 22, compared to 20% in untreated monarchs. Prior to death, butterflies exhibited signs of poisoning including uncoordinated flapping of wings and uncontrolled vibrating of wings and body. Imidacloprid did not reduce egg production. Shortened adult longevity has serious consequences for monarch population development, migration and overwintering. The potential widespread impact of imidacloprid-contaminated crop and wild flower nectar, may be a significant driver of monarch population decline. More research on the impact of neonicotinoid insecticides on the monarch and other butterflies should be viewed as a serious priority.

## 1. Introduction

The monarch butterfly, *Danaus plexippus* L., has suffered serious population decline in the eastern and western United States since the mid-1990s [1,2,3,4,5,6]. The causes of this decline are unclear but probably include multiple factors [7,8]. Larvae of monarchs feed only on milkweed (*Asclepias* spp.) which have been adversely impacted by the introduction, since the mid-1990s, of herbicide-resistant crops throughout much of the monarch’s North American range. Large areas of cropland where milkweeds survived are now largely milkweed-free, restricting monarch breeding [9,10,11]. The famed migration of monarchs [12] from northern tiers of North America to overwintering sites in central Mexico [13] and coastal California [14] may have also suffered from increased mortality during the migration [15]. Migrating monarchs need abundant nectar resources to fuel the migration and fall nectar resources may have declined during the past 25 years due to agricultural and urban development along migration routes and climate changes [16,17,18,19]. Another substantial change to the landscape since the mid-1990s has been the introduction, widespread use and environmental contamination by neonicotinoid insecticides [20].

Neonicotinoid insecticides have become the most widely used pesticides in the world [21]. Their success is partly due to their systemic nature which allows them to be applied in a variety of ways including seed dressings, soil drenches, tree injections and foliar sprays. After application the chemical moves throughout the plant tissues protecting the entire plant from insect pests. Neonicotinoids also have relatively low mammalian toxicity making them safer to use than the previously used organophosphate insecticides [21]. The first neonicotinoid insecticide, imidacloprid, was launched in 1991 and was the most widely used agrochemical in the world [21,22], until its ban in February 2018 by all member countries of the European Union [23]. Unfortunately, the impact of neonicotinoids extends beyond crop plants and target pests, bringing their use in agriculture and home gardens into question. Some early work indicated safety of neonicotinoids to beneficial arthropods [24,25], but overwhelmingly, adverse impacts on a wide range of beneficial insects including pollinators, have been reported [26]. To date, the majority of studies on the impact of neonicotinoids on non-target species have focused on honeybees and some bumblebee species. Neonicotinoid toxicity ranges from very low to very high in bees [27,28]. In addition, field-realistic concentrations of neonicotinoids have been reported to have substantial sub-lethal effects on bees including reduced ovary development [29], reduced reproductive success [30], impaired foraging [31,32], impaired avoidance of predators [32], impaired navigation [33] and reductions in learning and memory [34]. Sub-lethal effects may have substantial impacts on pollinator population level parameters [35] and neonicotinoids have been implicated in the global decline of pollinators [36]. Neonicotinoid-induced impairment of flight behavior and migratory ability have been described recently in a locust and a songbird [37,38]. Direct and indirect effects of neonicotinoids on non-target vertebrates have also been reported from rats, mice, rabbits, birds, frogs, fish, reptiles and deer [39,40]. Some recent studies reported that human prenatal and adult exposures to neonicotinoids are linked with Autism Spectrum Disorder, heart defects and neurological symptoms [20].

Exposure of pollinators to neonicotinoids may occur in a variety of ways. Some may be exposed to neonicotinoids applied as foliar sprays, although to protect bees there are restrictions on the use of some neonicotinoids at crop flowering [41]. Non-target arthropods may also be exposed to neonicotinoids as they forage on non-crop plants growing near agricultural areas [42,43]. Contamination of non-crop plants can occur from the dust produced during the drilling of treated seeds, which drifts onto surrounding vegetation [42,44]. The most likely route of pollinator exposure is uptake by the plant and dissemination through nectar and pollen [45]. In addition to crop flowers being contaminated with neonicotinoids, the mobility of these compounds in soil and water [28,41,46] means that residues can also be found in non-target plants at varying distances from crops [43,45]. Surveys of streams in agricultural and urban areas in the US have found that neonicotinoid residues are widespread in surface waters [47,48,49] and they have also been found in snow and spring meltwater in Canadian prairie wetlands [26]. Neonicotinoid insecticide residues in crop nectar and pollen differ considerably according to the amount applied to crops and landscapes as well as the method of application. Seed treatments result in relatively low levels, usually less than 10 ppb. Residues in pollen and nectar from crops treated with foliar applications, range from 10–100 ppb [50]. The greatest neonicotinoid residues (1000–4500 ppb) are found in nectar and pollen from landscape trees and plants treated with soil drenches [50]. Another less-researched but likely source of high levels of neonicotinoid contamination of honey and nectar, are home garden plants [51] which may receive application rates >40 times greater than used in agricultural systems. Garden plants propagated in nurseries receive even higher rates of application and may contain neonicotinoid residues in nectar and pollen of up to 45,000 ppb [50].

Studies on the impacts of neonicotinoids on butterflies are limited [52,53], despite two recent studies showing associations between butterfly population declines in Great Britain and California and the increase in use of neonicotinoid insecticides [54,55]. The few studies that have looked at the sublethal impact of neonicotinoids on butterflies have focused on the effects of acute exposure on larvae. Exposure of larvae of two British butterflies, the Large White (*Pieris brassicae* (L.)) and the Common Blue (*Polyommatus icarus* (Rottemburg)) to field-realistic levels of neonicotinoids during development, resulted in negative impacts. In *P. brassicae*, imidacloprid at 1–200 ppb significantly reduced pupal duration and the size of adult butterflies [56], while in *P. icarus*, clothianidin at 15 ppb significantly lengthened early instar development and reduced larval size [57]. The sublethal impact of field-realistic levels of imidacloprid and clothianidin on *D. plexippus* larvae, was reported by Pecenka and Lundgren [58] and Krischik et al. [50]. Clothianidin at levels of 0.5–5.0 ppb reduced weight and body length of first instar larvae. It also increased the period spent as first instar larvae and levels of 15.63 ppb resulted in 50% mortality of this instar [58]. Krischick et al. [50] also showed imidacloprid at 15 ppb significantly reduced survival of early instar larvae of *D. plexippus* with few surviving more than seven days. In contrast, the survival of adult *D. plexippus* and painted lady (*Vanessa cardui* (L.)) butterflies was not reduced when fed on imidacloprid-treated syrup or flowers at levels from 15–10,400 ppb. Fecundity and egg hatch rates in *D. plexippus* and *V. cardui* were similarly not affected by feeding on imidacloprid-treated syrup and flowers [50].

The paucity of data on the effect of neonicotinoids on adult butterflies including monarchs together with the current concern over the declining population of monarch butterflies especially in the western United States [6,8], prompted this brief laboratory study on the impact of a imidacloprid-contaminated diet on longevity and egg production in western monarch butterflies.

## 2. Materials and Methods 

Monarch butterflies used in this study were reared from eggs obtained by caging a wild-caught female with potted Showy Milkweed (*Asclepias speciosa* Torr.) at 28 °C under constant illumination. The female was obtained from a monarch/milkweed habitat near Paterson, Washington in August 2018. Sixty eggs were obtained over two days and the larvae reared on potted and cut *A. speciosa* in muslin cages held under the same conditions. The milkweed plants used in this study were obtained from an isolated home garden not subject to insecticide sprays, drift or run-off. Thus, it is unlikely larvae fed on these plants were contaminated with neonicotinoid insecticide. Forty adult butterflies (20 males, 20 females) eclosed during 25 September–1 October 2018 and were used in the experiment. On the day of eclosion butterflies were weighed (mg, Sartorius Ultra Micro Balance 4504 MP8), measured (mm, forewing) using calipers, and tested (by pressing clear adhesive tape (1.30 cm^2^) on the abdomen) for presence of the protozoan parasite, *Ophryocystis elektroscirrha* (OE) [59,60]. Tape samples were examined for OE at 63× magnification. Eleven males and eleven females were randomly assigned as the treatment group with the remaining nine males and nine females serving as the untreated or control group. All butterflies were marked with a unique coded adhesive tag to enable individual identification. Treated and untreated groups were held together in two muslin cages (60 × 35 × 35 cm) and stored at 28 °C under constant illumination for 22 days. The diet of both groups of butterflies was based on 5% sugar solution (made from granulated sugar), pure, or containing imidacloprid. Butterflies had ad libitum access to the sugar solution which was absorbed on four cotton wool feeding stations attached to all sides of the holding cage. Feeding stations were renewed daily. Butterflies were active in the cages, fed frequently at the feeding stations and received no other source of nourishment.

A commercial neonicotinoid concentrate formulation (Fruit, Citrus & Vegetable Insect Control, Bioadvanced, SBM Life Science Corp, Cary, NC, USA) containing 0.235% (2,350,000 ppb) imidacloprid was used to prepare the treated sugar diet. A treatment rate of 23.5 ppb was prepared by serial dilution with distilled water containing 5% sugar. This rate is a field-realistic level for imidacloprid and other neonicotinoids within the range reported in crop and wildflower nectar and pollen [43,45,50,56,57].

### 2.1. Longevity

Butterfly health and mortality was recorded daily in each group.

### 2.2. Oocyte Development

The effect of imidacloprid on oocyte development in *D. plexippus* was assessed by examining ovaries of 4–11 females from the untreated and treated groups at 12 and 22 days post-eclosion. Butterflies were removed from cages and placed in a freezer until dissection under a stereomicroscope. The abdomen of each female was opened ventrally under water and the paired ovaries were dissected out. Mature oocytes (chorionated) and immature oocytes (unchorionated) were counted.

### 2.3. Data Analysis

Students *t* tests were used to separate means for wet weights, forewing lengths and oocyte production.

## 3. Results

No evidence of OE was seen among the butterflies used in this experiment. Forewing lengths and wet weights at eclosion did not differ between treatments or sex except for a significantly lower mean weight of treated females vs. treated males (*t* test: *t* = 2.090, *p* = 0.049, 20 df) (Table 1). Forewing lengths of butterflies at 12 and 22 days post-eclosion did not differ between sex or treatment. After 12 days, wet weights of untreated and treated females and untreated males were comparable to their respective weights at eclosion. In contrast, treated males weighed significantly less after 12 days (*t* test: *t* = 3.383, *p* = 0.015, df 6) (Table 1). After 22 days, wet weights of untreated females were greater than at eclosion and day 12 but not significantly so. Insufficient numbers of treated individuals at day 22 prevented collection of wet weight data.

**Figure 1:**
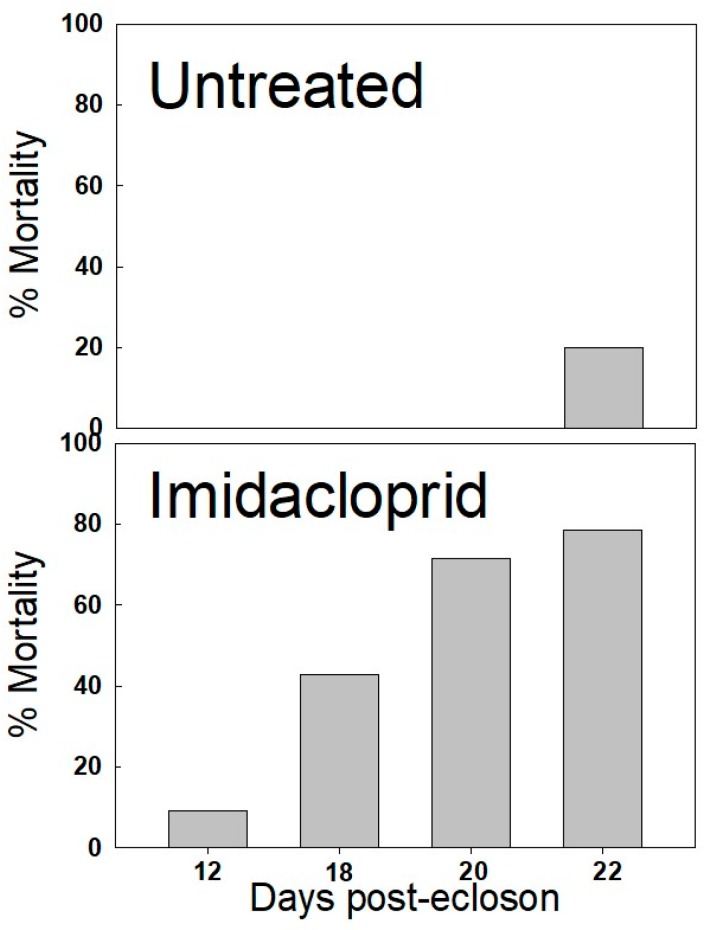
Mortality of caged monarch butterflies fed sugar water alone or sugar water containing 23.5 ppb imidacloprid during three weeks post-eclosion.

Monarch butterflies fed a sugar diet containing 23.5 ppb had substantially shorter longevity than those fed on a non-contaminated sugar diet. By day 22, only 3/14 individuals (21.4%) in the treated group were still alive compared to 8/10 (80%) in the untreated group. Mortalities in the treated group were recorded on days 12, 18, 20 and 22. In contrast, no mortalities occurred in the untreated group until day 22 (Figure 1). Butterflies in the treated group showed ill effects as early as day 12 when uncoordinated flapping of wings and uncontrolled vibrating of body and wings was recorded. These individuals were incapable of flight. Typically, these ill-effects lasted for 24–48 h before the butterfly died. None of these ill-effects were seen in untreated butterflies.

There was no difference in immature, mature or total oocyte production between treated and untreated female monarchs at 12 or 22 days (Table 2). Oocyte development was similar in treated and untreated females at 12 and 22 days post-eclosion with a mean of 102–115.5 oocytes/female at 12 days (*t* test: *t* = −0.614, *p* = 0.562, df 6) and 105.5–131.6/female at 22 days (*t* test: *t* = 1.092, *p* = 0.325, df 5) (Table 2).

## 4. Discussion

This brief and simple laboratory study while limited in scope and replication, indicates that the neonicotinoid, imidacloprid, incorporated in a sugar diet at a level within the range of concentrations reported from wild nectar, has a profound impact on the survival of adult monarch butterflies within a short period of time. Some mortality of treated monarchs occurred after 12 days and increased steadily, killing almost 80% of butterflies by day 22. Prior to death, treated monarchs displayed characteristic uncontrolled vibrating, trembling and flapping of wings, similar to neonicotinoid poisoning in honeybees [61]. Egg production by female monarchs was not affected by imidacloprid but clearly lifetime fecundity will be affected if females have shortened lives. Reproductive monarchs typically live for 4–6 weeks [12]. The level of imidacloprid chosen for incorporation in a sugar diet in this study (23.5 ppb) is at the lower end of the range of neonicotinoid levels found in the nectar of treated crops (10–100 ppb, [50]). The potential for exposure of nectar-foraging monarchs to much higher levels of neonicotinoids (up to 45,000 ppb) is great especially for those foraging on trees, bushes and ornamental plants in urban landscapes [50,62]. However, under natural conditions monarchs may not feed on contaminated nectar at every feeding as they were forced to in this study. Any reduction of adult longevity will have serious consequences for both breeding and non-breeding populations of monarchs. Reproductive females in spring-summer will lay fewer eggs over a shortened life-span, reducing population growth. Non-reproductive monarchs migrate in the fall and have an adult life span of 6–9 months [14], which is necessary to ensure successful overwintering and initiation of reproductive activity in spring. Nectar-borne imidacloprid consumed during migration or overwintering may result in monarchs not surviving the overwintering period.

Studies on the impact of neonicotinoids on butterflies are very limited compared to research on honeybees [52,53]. Only four studies have looked at neonicotinoids and butterfly species and three of these focused on larvae [56,57,58]. Only one study [50] looked at the impact of an imidacloprid-treated syrup-diet on adult butterflies. In this study, imidacloprid/syrup force-fed at 15 and 30 ppb, had no effect on the survival or fecundity of monarch and painted lady (*Vanessa cardui* L.) butterflies. In addition, free-ranging monarchs and painted ladies in small (60 × 60 × 120 cm) mesh cages that were allowed to feed on tropical milkweed (*Asclepias currassavica* L.) flowers containing 6030 ppb or 10,400 ppb, also showed no difference in survival and fecundity from non-exposed butterflies [50]. However, the authors of this study noted that monarchs may not have been able to forage adequately in these small cages. Uncontaminated 30% honey water sponges were also supplied as food in case nectar was limited in the heat of the day. It is possible that monarchs in these small cages may have fed less on the neonicotinoid-contaminated flowers than on the honey-water sponges. In contrast, the current study, which allowed no diet choice, showed a substantial negative impact of imidacloprid at 23.5 ppb incorporated in a sugar diet, on the survival of monarch butterflies. The current study used a liquid formulation of imidacloprid while Krischick et al. [50] used a granular formulation, a difference which may also have modified the impact of the compound on adult monarchs in the two studies.

Clearly, more work needs to be done on assessing the impact of nectar-borne neonicotinoids on butterfly and particularly monarch butterfly survival. This study highlights the likelihood that these ubiquitous insecticides are having a deleterious impact on butterfly populations. The nature of this impact requires more research on more butterfly species. While this study suggests that monarchs feeding on neonicotinoid-contaminated nectar have reduced longevity, this may not be the only impact. Although egg production appeared to be unaffected by exposure to imidacloprid, we do not know whether the viability of eggs and resultant larvae is unaffected. Mating behavior could also be affected as recently shown in a parasitic wasp species [63]. The monarch butterfly depends on continental-scale fall migration to escape from winter climates in mid-high latitudes and recolonization of these areas the following spring [14]. Monarch migration is powered by feeding on a wide range of flower nectar in natural, agricultural and urban landscapes, some of which is likely to be contaminated with neonicotinoid residues. Could the migratory ability of monarchs be affected by these residues? Neonicotinoid-mediated impairment of foraging behavior has been reported for bumblebees [32,64,65]. Flight behavior of locusts (*Locusta migratoria* L.) is impaired by imidacloprid [37] and the migration of a songbird is also impaired by this compound [38]. The possible impact of sub-lethal nectar-borne neonicotinoids on monarch migration should be investigated as a matter of urgency. The monarch may be exposed to neonicotinoids in two of four developmental stages (larvae and adults), thus sub-lethal effects may be additive. Neonicotinoid-exposed monarch larvae show developmental problems [58], which may exacerbate neonicotinoid-mediated reduction in adult longevity.

The serious declines in western and eastern US monarch populations recorded over the past two decades likely have a multifactorial cause [7]. The widespread and increased use of neonicotinoids and other insecticides is just one part of a complex situation. However, the increasing body of knowledge on the subtle yet chronic impacts of neonicotinoids on invertebrates and vertebrates, suggests that insecticides may be a significant driver of monarch butterfly decline. If monarchs are being impacted by neonicotinoids, then undoubtedly other butterfly species are also being affected, which may explain the large-scale decline of butterflies reported in the United Kingdom [54] and California [55]. Monitoring butterfly populations over the next 5–10 years in the United Kingdom and Europe, where the use of neonicotinoid insecticides has recently been banned [23], may provide field evidence for the adverse effects of this class of pesticides on butterfly populations.

## 5. Conclusions

Newly-eclosed monarch butterflies fed ad libitum a sugar solution containing 23.5 ppb of the neonicotinoid insecticide, imidacloprid, which is within the range of neonicotinoid concentrations found in nectar, had reduced longevity, suffering 78.8% mortality 22 days post-eclosion, compared to 20% mortality in butterflies fed sugar-only. Butterflies fed on the imidacloprid-laced diet invariably took 2–3 days to die following ill-effects manifested as uncoordinated flapping of wings and uncontrolled vibrating of wings and body. Imidacloprid did not have a direct impact on egg production in treated monarchs, although fecundity of these monarchs would be reduced by virtue of reduced longevity. Shortened adult longevity has serious consequences for monarch population development, migration and overwintering and the potential widespread impact of imidacloprid-contaminated crop and wild flower nectar, may be a significant driver of monarch population decline. More research on the impact of neonicotinoid insecticides on the biology and ecology of monarch and other butterflies should be viewed as a serious priority

## Figures and Tables

**Table 1:** Forewing lengths (mm) and wet weights (mg) of untreated and imidacloprid-treated monarch butterflies at eclosion and 12 and 22 days post-eclosion. Values in rows with a different letter are significantly different from corresponding value for other sex (*p* < 0.05). Value with asterisk is significantly different from corresponding value at eclosion (*p* < 0.05). Numbers in parentheses represent number of individuals examined.

Forewing (mm)	Male	Female	Wet Weight (mg)	Male	Female
**Eclosion**					
Untreated	46.0 ± 1.1 ^a^ (9)	47.2 ± 2 ± 0.7 ^a^ (9)	Untreated	413.2 ± 18.0 ^a^ (9)	422.8 ± 28.7 ^a^ (9)
Treated	46.2 ± 0.5 ^a^ (11)	45.1 ± 0.7 ^a^ (11)	Treated	466.8 ± 28.7 ^a^ (11)	389.0 ± 23.7 ^b^ (11)
**12 days post-eclosion**					
Untreated	48.0 ± 0.8 ^a^ (4)	46.8 ± 0.8 ^a^ (4)	Untreated	467.5 ± 15.2 ^a^ (4)	423.5 ± 31.9 ^a^ (4)
Treated	46.0 ± 0.4 ^a^ (4)	46.0 ± 1.1 ^a^ (4)	Treated	377.2 ± 21.9 ^a^ * (4)	385.2 ± 59.2 ^a^ (4)
**22 days post-eclosion**					
Untreated	46.7 ± 1.2 ^a^ (3)	47.6 ± 1.1 ^a^ (5)	Untreated	360.0 ± 24.2 ^a^ * (3)	457.8 ± 23.9 ^b^ (5)
Treated		46.0 ± 2.0 (2)	Treated		466.5 ± 44.5 (2)

**Table 2:** Oocyte production in untreated and imidacloprid-treated monarch butterflies at 12 and 22 days post-eclosion. Numbers in parentheses represent number of individuals examined.

Post-Eclosion Day and Treatment	No. Immature Oocytes	No. Mature Oocytes	No. Total Oocytes	*t* Test on Total Oocytes (Treated *v* Untreated)
12				
Untreated	55.8 ± 13.1	59.8 ± 6.4	115.5 ± 19.3 (4)	
Treated	42.8 ± 4.2	59.2 ± 9.8	102.0 ± 10.5 (4)	*t* = −0.614, 6 df, *p* = 0.562
22				
Untreated	46.0 ± 6.2	85.6 ± 7.0	131.6 ± 10.2 (5)	
Treated	37.0 ± 9.0	68.5 ± 22.5	105.5 ± 31.5 (2)	*t* = −1.092, 5 df, *p* = 0.325

## References

[1] Brower L.P., Taylor O.R., Williams E.H., Slayback D.A., Zubieta R.R., Ramirez R.R. (2012). Decline of monarch butterflies overwintering in Mexico: Is the migratory phenomenon at risk?. Insect Conserv. Divers..

[2] Semmens B.X., Semmens D.J., Thogmartin W.E., Wiederholt R., Lopez-Hoffman L., Diffendorfer J.E., Pleasants J.M., Oberhauser K.M., Yatlor O.R. (2016). Quasi-extinction risk and population targets for the eastern migratory population of monarch butterflies (*Danaus plexippus*). Sci. Rep..

[3] Stenoien C., Nail K.R., Zalucki J.M., Parry H., Oberhauser K.S., Zalucki M.P. (2016). Monarchs in decline: A collateral landscape-level effect of modern agriculture. Insect Sci..

[4] Marini L., Zalucki M.P. (2017). Density dependence in the declining population of the monarch butterfly. Sci. Rep..

[5] Pleasants J.M., Zalucki M.P., Oberhauser K.S., Brower L.P., Taylor O.R., Thogmartin W.E. (2017). Interpreting surveys to estimate the size of the monarch butterfly population: Pitfalls and prospects. PLoS ONE.

[6] Schultz C.B., Brown L.M., Pelton E., Crone E.E. (2017). Citizen science monitoring demonstrates dramatic declines of monarch butterflies in western North America. Biol. Conserv..

[7] Thogmartin W.E., Lopez-Hoffman L., Rohweder J., Diffendorfer J., Drum R., Semmens D. (2017). Restoring monarch butterfly habitat in the Midwestern US: All hands on deck. Environ. Res. Lett..

[8] Pelton E., Schultz C.B., Jepsen S.J., Hoffman Black S., Crone E.E. (2019). Western monarch population plummets: Status, probable causes and recommended conservation actions. Front. Ecol. Evol..

[9] Hartzler R.G. (2010). Reduction in common milkweed (*Asclepias syriaca*) occurrence in Iowa cropland from 1999 to 2009. Crop Prot..

[10] Pleasants J.M., Oberhauser K.S. (2013). Milkweed loss in agricultural fields because of herbicide use: Effect on the monarch butterfly population. Insect Conserv. Divers..

[11] Zaya D.N., Pearse L.S., Spyreas G. (2017). Long term trends in Midwestern milkweed abundances and their relevance to monarch butterfly declines. Bioscience.

[12] Brower L.P. (1995). Understanding and misunderstanding the migration of the monarch butterfly (Nymphalidae) in North America. J. Lep. Soc..

[13] Wassenaar L.L., Hobson K.A. (1998). Natal origins of migratory monarch butterflies at wintering colonies in Mexico: New isotopic evidence. Proc. Natl. Acad. Sci. USA.

[14] James D.G., James T.S., Seymour L., Kappen L., Russell T., Harryman B., Bly C. (2018). Citizen scientist tagging reveals destinations of migrating monarch butterflies, *Danaus plexippus* (L.) from the Pacific Northwest. J. Lep. Soc..

[15] Inamine H., Ellner S.P., Springer J.P., Agrawal A.A. (2016). Linking the continental migratory cycle of the monarch butterfly to understand its population decline. Oikos.

[16] Badgett G., Davis A.K. (2015). Population trends of monarchs at a northern monitoring site: Analyses of 19 years of fall migration counts at Peninsula Point, MI. Ann. Entomol. Soc. Am..

[17] Malcolm S.B. (2016). Anthropogenic impacts on mortality and population viability of the monarch butterfly. Ann. Rev. Ent..

[18] Agrawal A.A., Inamine H. (2018). Mechanisms behind the monarch’s decline. Science.

[19] Tracey J.L., Kantola T., Baum K.A., Coulson R.N. (2019). Modeling fall migration pathways and spatially identifying potential migratory hazards for the eastern monarch butterfly. Landscape Ecol..

[20] Craddock H.A., Huang D., Turner P.C., Quiros-Alcala L., Payne-Styrges D.C. (2019). Trends in neonicotinoid pesticide residues in food and water in the United States, 1999–2015. Env. Health.

[21] Jeschke P., Nauen R., Schindler M., Elbert A. (2011). Overview of the status and global strategy for neonicotinoids. J. Agric. Food Chem..

[22] Pollak P. (2011). Fine Chemicals: The Industry and the Business.

[23] Blake R. (2018). EU neonicotinoid ban removes vital tools in global fight against pests. Outlooks Pest Manag..

[24] Mizell R.F., Sconyers M.C. (1992). Toxicity of imidacloprid to selected arthropod predators in the laboratory. Fla. Entomol..

[25] James D.G. (1997). Imidacloprid increases egg production in *Amblyseius victoriensis* (Acari: Phytoseiidae). Exp. Appl. Acarol..

[26] Main A.R., Webb E.B., Goyne K.W., Mengel D. (2018). Neonicotinoid insecticides negatively affect performance measures of non-target terrestrial arthropods: A meta-analysis. Ecol. Appl..

[27] Isawa T., Motoyama N., Ambrose J.T., Roe R.M. (2004). Mechanism for the differential toxicity of neonicotinoid insecticides in the honey bee, *Apis mellifera*. Crop Prot..

[28] Casida J.E. (2018). Neonicotinoids and other insect nicotinic receptor competitive modulators: Progress and prospects. Ann. Rev. Entomol..

[29] Baron G.L., Raine N.E., Brown M.J.F. (2017). General and species-specific impacts of a neonicotinoid insecticide on the ovary development and feeding of wild bumblebee queens. Proc. R. Soc. B Biol. Sci..

[30] Whitehorn P.R., O’Connor S., Wackers F.L., Goulson D. (2012). Neonicotinoid pesticide reduces bumble bee colony growth and queen production. Science.

[31] Feltham H., Park K., Goulson D. (2014). Field realistic doses of pesticide imidacloprid reduce bumblebee pollen foraging efficiency. Ecotoxicology.

[32] Tan K., Chen W., Dong S., Liu X., Wang Y., Nish J.C. (2014). Imidacloprid alters foraging and decreases bee avoidance of predators. PLoS ONE.

[33] Henry M., Beguin M., Requier F., Rollin O., Odoux J.F., Aupinel P. (2012). A common pesticide decreases foraging success and survival in honeybees. Science.

[34] Stanley D.A., Smith K.E., Raine N.E. (2015). Bumblebee learning and memory are impaired by chronic exposure to a neonicotinoid pesticide. Sci. Rep..

[35] Woodcock B.A., Isaac N.J., Bullock J.M., Roy D.B., Garthwaite D.G., Crowe A., Pywell R.F. (2016). Impacts of neonicotinoid use on long-term population changes in wild bees in England. Nat. Commun..

[36] Van der Sluijs J.P., Amaral-Rogers V., Belzunces L.P., Bijleveld van Lexmond M.F.I.J., Bonmatin J.M., Chagnon M., Downs C.A., Furlan L., Gibbons D.W., Glorio C. (2015). Conclusions of the worldwide integrated assessment on the risks of neonicotinoids and fipronil to biodiversity and ecosystem functioning. Environ. Sci. Pollut. Res..

[37] Parkinson R.H., Gray J.R. (2019). Neural conduction, visual motion detection and insect flight behavior are disrupted by low doses of imidacloprid and its metabolites. Neurotoxicology.

[38] Eng M.L., Stutchbury B.J.M., Morrissey C.A. (2017). Imidacloprid and chlorpyrifos insecticides impair migratory ability in a seed-eating songbird. Sci. Rep..

[39] Gibbons D., Morrissey C., Mineau P. (2015). A review of the direct and indirect effects of neonicotinoids and fipronil on vertebrate wildlife. Environ. Sci. Pollut. Res..

[40] Berheim E.H., Jenks J.A., Lundgren J.G., Michel E.S., Grove D., Jensen W.F. (2019). Effects of neonicotinoid insecticides on physiology and reproductive characteristics of captive female and fawn white-tailed deer. Sci. Rep..

[41] Godfray H.C.J., Blacquiere T., Field L.M., Halls R.S., Potts S.G., Raine N.E., Vanbergen A.J., McLean A.R. (2014). A restatement of recent advances in the natural science evidence base concerning neonicotinoid insecticides and insect pollinators. Proc. R. Soc. B.

[42] Krupke C.H., Hunt G.J., Eitzer B.D., Andino J., Given K. (2012). Multiple routes of pesticide exposure for honeybees living near agricultural fields. PLoS ONE.

[43] Botias C., David A., Hill E.M., Goulson D. (2016). Contamination of wild plants near neonicotinoid-treated crops, and implications for non-target insects. Sci. Total Environ..

[44] Limay-Rios V., Forero L.G., Xue Y., Smith J., Baute T., Schaafsma A. (2016). Neonicotinoid insecticide residues in soil dust and associated parent soil in fields with a history of seed treatment use on crops in southwestern Ontario. Environ. Toxicol. Chem..

[45] David A., Botias C., Abdul-Sada A., Nicholls E., Rotheray E.L. (2016). Widespread contamination of wildflower and bee-collected pollen with complex mixtures of neonicotinoids and fungicides commonly applied to crops. Env. Int..

[46] Douglas M.R., Rohr J.R., Tooker J.F. (2014). Neonicotinoid insecticide travels through a soil food chain, disrupting biological control of non-target pests and decreasing soya bean yield. J. Appl. Ecol..

[47] Hladik M.L., Kolpin D.W., Kuivila K.M. (2014). Widespread occurrence of neonicotinoid insecticides in streams in a high corn and soybean producing region, USA. Environ. Pollut..

[48] Hladik M.L., Kolpin D.W. (2015). First national scale reconnaissance of neonicotinoid insecticides in streams across the USA. Environ. Chem..

[49] Morrissey C.A., Mineau P., Devries J. (2015). Neonicotinoid contamination of global surface waters and associated risk to aquatic invertebrates: A review. Environ. Int..

[50] Krischik V., Rogers M., Gupta G., Varshney A. (2015). Soil-applied imidacloprid translocates to ornamental flowers and reduces survival of adult *Coleomegilla maculata*, *Harmonia axyridis* and *Hippodamia convergens* lady beetles, and larval *Danaus plexippus* and *Vanessa cardui* butterflies. PLoS ONE.

[51] Keim B. (2012). Backyard Pesticide Use May Fuel Bee Die-Offs. www.wired.com/2012/04/neonicotinoids-gardens/.

[52] Mule R., Sabella G., Robba R., Manachini B. (2017). Systematic review of the effects of chemical insecticides on four common butterfly families. Front. Environ. Sci..

[53] Braak N., Neve R., Jones A.K., Gibbs M., Breuker C.J. (2018). The effects of insecticides on butterflies: A review. Environ. Pollut..

[54] Gilburn A.S., Bunnefeld N., McVean Wilson J., Botham M.S., Brereton T.M., Fox R., Goulson D. (2015). Are neonicotinoid insecticides driving declines of widespread butterflies?. Peer J..

[55] Forrister M.L., Cousens B., Harrison J.G., Anderson K., Thorne J.H., Waetjen D., Nice C.C., De Parsla M., Hladik M.L., Meese R. (2016). Increasing neonicotinoid use and the declining butterfly fauna of lowland California. Biol. Lett..

[56] Whitehorn P., Norville G., Gilburn A., Goulson D. (2018). Larval exposure to the neonicotinoid imidacloprid impacts adult size in the farmland butterfly *Pieris brassicae*. Peer J..

[57] Basley K., Goulson D. (2018). Effects of field-relevant concentrations of clothianidin on larval development of the butterfly *Polyommatus icarus* (Lepidoptera: Lycaenidae). Environ. Sci. Technol..

[58] Pacenka J.R., Lundgren J.G. (2015). Non-target effects of clothianidin on monarch butterflies. Sci. Nat..

[59] McLaughlin R.E., Myers J. (1970). *Ophryocystis elektroscirrha* sp. n., a neogregarine pathogen of the monarch butterfly *Danaus plexippus* (L.) and the Florida queen butterfly *D. gilippus berenice* Cramer. J. Protozool..

[60] Altizer S.M., Oberhauser K.S., Brower L.P. (2000). Association between host migration and the prevalence of a protozoan parasite in natural populations of monarch butterflies. Ecol. Entomol..

[61] Suchail S., Guez D., Belzunces L.P. (2000). Characteristics of imidacloprid toxicity in two *Apis mellifera* subspecies. Environ. Tox. Chem..

[62] Hopwood J., Shepherd M. (2012). Neonicotinoids in your garden. Wings Fall.

[63] Kremer A.N., King B.H. (2019). A neonicotinoid affects the mating behavior of *Spalangia endius* (Hymenoptera: Pteromalidae), a biological control agent of filth flies. Environ. Entomol..

[64] Gill R.J., Raine N.E. (2014). Chronic impairment of bumblebee natural foraging behavior induced by sublethal pesticide exposure. Funct. Ecol..

[65] Lamsa J., Kuusela E., Tuomi J., Juntunen S., Watts P.C. (2018). Low dose of neonicotinoid insecticide reduces foraging motivation of bumblebees. Proc. R. Soc. B.

